# Trainee-led research using an integrated knowledge translation or other research partnership approaches: a scoping reviews

**DOI:** 10.1186/s12961-021-00784-0

**Published:** 2021-11-02

**Authors:** Christine E. Cassidy, Hwayeon Danielle Shin, Emily Ramage, Aislinn Conway, Kelly Mrklas, Celia Laur, Amy Beck, Melissa Demery Varin, Sandy Steinwender, Tram Nguyen, Jodi Langley, Rachel Dorey, Lauren Donnelly, Ilja Ormel

**Affiliations:** 1 School of Nursing, Dalhousie University, Halifax, NS Canada; 2 School of Health Sciences, University of Newcastle, Callaghan, Australia; 3 Better Outcomes and Registry Network (BORN), Ottawa, ON Canada; 4 Alberta Health Services Foothills Medical Centre, Calgary, AB Canada; 5 Institute for Health System Solutions and Virtual Care, Women’s College Hospital, Toronto, Canada; 6 Faculty of Nursing, University of Calgary, Calgary, AB Canada; 7 School of Nursing, University of Ottawa, Ottawa, ON Canada; 8 Health Information Science, Western University, London, ON Canada; 9 School of Epidemiology and Public Health, University of Ottawa, Ottawa, ON Canada; 10 School of Health and Human Performance, Dalhousie University, Halifax, NS Canada; 11 Dalhousie University, Halifax, NS Canada; 12 Department of Family Medicine, McGill University, Montreal, QC Canada

**Keywords:** Integrated knowledge translation, Health research, Research trainees, Partnership approaches, Collaborative research

## Abstract

**Background:**

There are increasing expectations for researchers and knowledge users in the health system to use a research partnership approach, such as integrated knowledge translation, to increase the relevance and use of research findings in health practice, programmes and policies. However, little is known about how health research trainees engage in research partnership approaches such as IKT. In response, the purpose of this scoping review was to map and characterize the evidence related to using an IKT or other research partnership approach from the perspective of health research trainees in thesis and/or postdoctoral work.

**Methods:**

We conducted this scoping review following the Joanna Briggs Institute methodology and Arksey and O’Malley’s framework. We searched the following databases in June 2020: MEDLINE, Embase, CINAHL and PsycINFO. We also searched sources of unpublished studies and grey literature. We reported our findings in accordance with the Preferred Reporting Items for Systematic Reviews and Meta-Analyses Extension for Scoping Reviews.

**Results:**

We included 74 records that described trainees’ experiences using an IKT or other research partnership approach to health research. The majority of studies involved collaboration with knowledge users in the research question development, recruitment and data collection stages of the research process. Intersecting barriers to IKT or other research partnerships at the individual, interpersonal and organizational levels were reported, including lack of skills in partnership research, competing priorities and trainees’ “outsider” status. We also identified studies that evaluated their IKT approach and reported impacts on partnership formation, such as valuing different perspectives, and enhanced relevance of research.

**Conclusion:**

Our review provides insights for trainees interested in IKT or other research partnership approaches and offers guidance on how to apply an IKT approach to their research. The review findings can serve as a basis for future reviews and primary research focused on IKT principles, strategies and evaluation. The findings can also inform IKT training efforts such as guideline development and academic programme development.

**Supplementary Information:**

The online version contains supplementary material available at 10.1186/s12961-021-00784-0.

## Introduction

Collaborative research approaches, such as coproduction, codesign, engaged scholarship and integrated knowledge translation (IKT) [1], aim to produce relevant research findings to address healthcare issues. IKT, specifically, focuses on making research more useful through research partnerships. IKT is defined as “a model of collaborative research, where researchers work with knowledge users who identify a problem and have the authority to implement the research recommendations” [2]. Studies have shown that use of an IKT approach improves the quality of research [3], enhances value for research among decision-makers [4], increases capacity among decision-makers for engaging in research [4–6], and creates more impactful and useful research findings [3, 7, 8]. Research partnerships have been shown to be critical during the COVID-19 pandemic, as patients, citizens, healthcare providers, researchers, policy-makers and health system leaders from around world have come together to collectively address this global crisis [9].

Despite the value of IKT and other research partnership approaches, studies report many challenges in establishing and maintaining research partnerships with knowledge users [3, 4, 10]. Significant time is needed to develop trusting, authentic relationships, and there may be insufficient resources to support partnership development and maintenance [10]. Further, differing needs and priorities among researchers and health system decision-makers [3, 4, 10], as well as unclear goals, roles and expectations, can hinder research partnerships [4].

Health system leaders have identified a lack of researcher preparation for engaging in collaborative partnerships as a significant barrier to successful research partnerships [11]. This can lead to ineffective researcher behaviour (e.g., mismatch of researcher interests and organizational needs, lack of researcher understanding of health system context, lack of respect) and affect the development of positive, mutually beneficial research partnerships [11]. Other studies have shown that researchers require specific knowledge and skills for working in partnership with health system decision-makers [12]. However, researchers often do not have the opportunity to learn how to establish effective research partnerships with knowledge users in the health system [10].

Specific training in IKT or other research partnerships is needed to promote collaborative health research moving forward. Most graduate students do not receive formal training in collaborative health research approaches [13, 14]. Efforts are needed to support trainees, defined as graduate students (master’s, doctoral) and postdoctoral fellows, in developing an understanding of health system context and skills to engage in collaborative research partnerships [11, 15]. Such training efforts are essential to support trainees in building trusting, effective relationships with knowledge users to foster meaningful, ethical research with relevant outcomes [16].

Currently, the IKT or research partnership literature describes strategies, barriers and facilitators to research partnerships from the perspective of researchers and knowledge users [10, 11, 17–19]. However, little is known about how trainees engage in research partnership approaches such as IKT. This is needed as a first step to inform research trainees on the use of IKT or other research partnership approaches and future academic training modernization efforts. As such, the purpose of this scoping review was to map and characterize the evidence related to using an IKT or other research partnership approach from the perspective of health research trainees in thesis and/or postdoctoral work.

## Methods

We conducted this scoping review following the Joanna Briggs Institute (JBI) methodology [20, 21] and Arksey and O’Malley's framework [22]. Our a priori protocol has been published previously [23]. This full report followed the Preferred Reporting Items for Systematic Reviews and Meta-Analysis Extension for Scoping Reviews (PRISMA-ScR) checklist [24] (Additional File 2).

### Stage 1: Identifying the research question

We conducted a scoping review to map and characterize the available evidence related to using an IKT approach or other research partnership approach from the perspectives of trainees in thesis and/or postdoctoral work. Specifically, we answered the following research question:

1. How have IKT or other research partnership approaches been applied in thesis and/or postdoctoral health research?

Additional research objectives included:

- Identifying IKT/research partnership principles, strategies and/or tools used in trainee- led health research
- Identifying barriers and facilitators to using IKT or other research partnership approaches in trainee-led health research
- Identifying if/how outcomes were reported and evaluated in trainee-led health research using IKT or other research partnership approaches.

### Stage 2: Identifying relevant studies

#### Participant

This review considered literature for which health research trainees (i.e., graduate students [master’s, doctoral] and postdoctoral fellows) were the primary author/researcher of the paper. Postdoctoral fellows were described as postdoctoral researchers or postdoctoral research associates. Students/fellows in the position of a trainee meant that the included studies were related to the student’s or fellow’s thesis/programme project/dissertation/fellowship projects.

#### Concept

This review considered studies that explored IKT or other research partnership approaches in trainee-led health research. We included studies that described the trainee’s experience with IKT research or related partnership approaches (i.e., manuscript or thesis/dissertation chapter that provides a reflection or description of the approach, text or opinion paper describing how the partnership approach was used). Included papers explained how the research partnerships approach was used, including principles, strategies and/or tools. Studies that stated the use of a research partnership approach but did not describe how it was used were excluded. Studies that described barriers and facilitators to using an IKT or other research partnership approach were included. Studies that evaluated the IKT approach were also included. Papers that did not describe a research study were excluded as wrong design. For this review, we used the following operational terms and definitions (Table 1).

**Table 1:** Operational terms and definitions

Term	Definition
Integrated knowledge translation	“A model of collaborative research, where researchers work with knowledge users who identify a problem and have the authority to implement the research recommendations” [2]
Research partnerships	“Individuals, groups, or organizations engaged in collaborative research activity involving at least one researcher (e.g., individual affiliated with an academic institution) and any stakeholder actively engaged in any part of the research process (e.g., decision or policy-maker, health care administrator or leader, community agency, charities, network, patients, lived experience advisor, etc.)” [144]
Approaches	The IKT and research partnership activities that comprise or promote collaboration in the research process
Barrier	“A circumstance or obstacle that keeps people or things apart or prevents communication or progress” [145]
Facilitator	“Someone or something that facilitates (to make easier)” [146]

#### Context

We considered literature focused on trainee-led health research. For the purpose of this review, health research referred to research that aimed to “increase our knowledge of health, disease, and health services, and to then apply that knowledge to help people lead healthier lives” [25]. It also included “biomedical research, epidemiological studies, and health services research, as well as studies of behavioral, social, and economic factors that affect health” [26].

### Search strategy

In collaboration with a health science librarian, we developed a search strategy to locate both published and unpublished primary studies, reviews, and text and opinion papers. Hoekstra and colleagues’ [27] comprehensive search strategy for synthesizing the research partnership literature was used to inform our search strategy. We followed the three-step process in accordance with the JBI Scoping Review Methodology. First, we conducted an initial limited search of MEDLINE and CINAHL to identify articles on the topic. Second, from the selected articles, we derived relevant text words and index terms to develop a full search strategy. Third, the search strategy, including all identified keywords and index terms, was adapted for all included information sources. We searched the following databases on 24 June 2020: MEDLINE (Ovid), Embase (Elsevier), CINAHL (EBSCO) and PsycINFO (EBSCO). The final search strategy for each database can be found in Additional file 1. We included peer-reviewed studies, editorials and commentaries. We also searched sources of unpublished studies and grey literature (not empirical studies) including ProQuest Dissertation & Theses Global databases (ProQuest) and the first 50 pages of Google Scholar during the period of September–November 2020. Only papers published in English were included. No date limits were applied, to allow for exploration of the use of IKT or other research partnership approaches in trainee-led research over time. We also used websites of research and academic institutions and health system organizations, together with concept papers, reports and blog posts, that reported non-peer-reviewed literature. We searched relevant websites of professional bodies or organizations such as the Integrated Knowledge Translation Research Network (IKTRN), Strategy for Patient-Oriented Research (SPOR) SUPPORT [Support for People and Patient-Oriented Research and Trials] Units, National Institutes of Health (NIH) Dissemination & Implementation, National Institute for Health Research (NIHR) Collaborations for Leadership in Applied Health Research and Care (CLAHRCs), and KT Canada, and we also contacted individuals or groups for additional material (i.e., Twitter, IKTRN members, relevant KT listservs). We used our professional networks in the area of IKT and collaborative health research to email relevant content experts to identify additional sources that met the inclusion criteria. Reference chaining was conducted with all included papers.

### Stage 3: Study selection

All identified citations were collated and uploaded into Covidence [28], and duplicates were automatically removed. A pair of reviewers (CC, HDS, JL, CL, ER, KM, MDV) independently screened and assessed titles and abstracts against the inclusion criteria. Next, full-text articles were retrieved for potentially relevant studies. After screening titles and abstracts, two independent reviewers (CC, HDS, JL, CL, ER, KM, MDV) assessed the full text of relevant studies in detail against the inclusion criteria. Any discrepancies between the reviewers at each stage of the study selection process were resolved through discussion or by a third reviewer. We sought out the trainee status of each primary author during the full-text assessment stage. When papers did not mention a primary author’s status, we searched the author’s name and the indicated affiliation in Google. If the primary author was found to be a trainee, we included the paper. We also made use of available LinkedIn profiles of the authors to identify their trainee status.

### Stage 4: Charting data

We developed a data extraction tool to capture information on the general characteristics of the included paper, trainees’ characteristics, IKT approaches, the trainee-reported barriers and facilitators to knowledge user engagement, and outcomes. Two reviewers (CC, HDS) first pilot-tested the extraction tool on three studies to identify any discrepancies and ensure consistency of data extraction. Then reviewers (CC, HDS, JL, TN, ER, AB, AC, RD, LD, MDV) were paired and independently extracted data using Covidence. Conflicts between reviewers regarding data extracted were resolved through discussion or by a third reviewer.

We used several frameworks to analyse the extracted data. First, we mapped included papers onto the seven phases of the knowledge-to-action (KTA) cycle [29] based on the reported research purpose and objectives. Second, we categorized the knowledge users’ engagement into the five levels of public participation of the International Association for Public Participation (IAP2) spectrum to help conceptualize the data and offer a structure for reporting the results [30]. Third, we identified the research stages [31] that knowledge users were engaged in, including (1) development of research question, (2) development of research proposal, (3) pre-study launch administration, (4) recruitment and data collection, (5) data analysis, and (6) dissemination and implementation. Fourth, we used the Workgroup for Intervention Development and Evaluation Research (WIDER) [32] reporting checklist to describe details about the IKT approach, including (1) content (nature and goal of the study and/or IKT partnership); (2) mode of delivery (specific types of IKT activities in which knowledge users were involved); (3) duration and/or frequency (timing of IKT activities); (4) participants (who was involved in specific IKT activities); and (5) personnel (who coordinated or led IKT activities) (review objective A). Fifth, we used the Capability, Opportunity, and Motivation-Behaviour (COM-B) model [33] and Theoretical Domains Framework (TDF) [34] to describe trainee-reported barriers and facilitators to knowledge user engagement (review objective B). We first coded data using the COM-B and TDF, and then generated themes inductively. Next, we used McLeroy’s social-ecological model [35] to map where the reported barriers and facilitators existed within the trainee’s research ecosystem (i.e., individual, interpersonal and/or organizational levels). Lastly, we developed three categories informed by the IKT outcomes reported by Gagliardi [4, 36] to organize reported outcomes and impact of the IKT approach in the included studies (review objective C), if the studies reported outcomes. We coded reported outcomes into the following three categories: (1) immediate outcome of partnership formation (e.g., mutual understanding of language, work style, needs and constraints, and appreciation for the collaborative process are established [4]); (2) intermediate outcome of partnership that occurred during the preparation and research process (e.g., identification of research questions, and conduct of research[36]); and (3) long-term outcome of partnership at the completion of the research process and post-study (e.g., scale-up/spread of research, and use of research in practice and policy [4, 36].)

One reviewer (HDS) initially coded data using the coding schemes for the first five papers. This coding was verified by a second reviewer (CC) to identify any discrepancies and ensure consistency in coding. After verifying the coding strategy, one reviewer (HDS) coded the remaining data, and the second reviewer (CC) verified the coded data. Findings were presented and reviewed with all team members to interpret key findings.

### Stage 5: Collating, summarizing and reporting results

We charted the data in a tabular form to align with the review objectives. In addition to the tables, we created a figure of the barriers and facilitators. We also produced descriptive numerical summaries of the quantitative data (i.e., frequency counts). Lastly, we provided a narrative summary to accompany these presentations and described how the findings addressed the review's question and objectives.

## Results

**Fig. 1:**
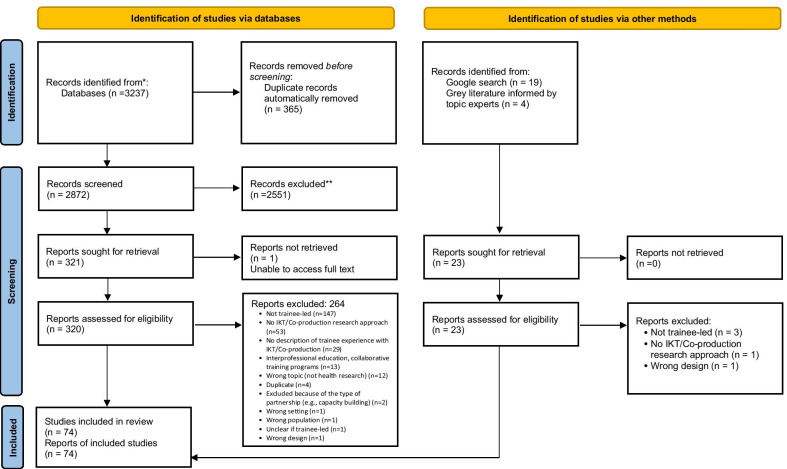
PRISMA flow chart. From: Page MJ, McKenzie JE, Bossuyt PM, Boutron I, Hoffmann TC, Mulrow CD, et al. The PRISMA 2020 statement: an updated guideline for reporting systematic reviews. BMJ 2021;372:n71. https://doi.org/10.1136/bmj.n71. For more information, visit: http://www.prisma-statement.org

Our database searches resulted in 3237 citations. We identified an additional 23 relevant papers through other information sources (e.g., IKTRN, Google Search), for a total of 3260 citations. After duplicate removal, 2895 citations remained for assessment against the inclusion criteria. After screening titles and abstracts, 343 citations remained for full-text review, and 74 citations, describing 72 studies, were included. See Fig. 1 for the PRISMA flow chart.

### Characteristics of included studies and trainees

Of the 74 papers, 34 were peer-reviewed studies, 28 were theses, 9 were web-based sources and 3 were other published grey literature. The 72 studies were a mix of participatory action research (*n* = 41), mixed or multi-methods (*n* = 12), codesign (*n* = 1), scoping or systematic review (*n* = 2) or other qualitative designs (*n* = 12). Web-based sources included casebooks from the IKTRN and text interviews of trainees who used an IKT approach in their research. Most grey literature sources were theses (i.e., primary research) and non-peer-reviewed journal articles.

Papers originated from Canada (*n* = 36), the United States (*n* = 23), Australia (*n* = 5), the United Kingdom (*n* = 6), Norway (*n* = 1), Slovenia (*n* = 1) and Iceland (*n* = 1). The trainees (primary authors of these papers) were in nursing (*n* = 14), education (*n* = 12), public or population health (*n* = 8), medicine (*n* = 5), social work (*n* = 5), physiotherapy (*n* = 2), occupational health (*n* = 3), dietetics (*n* = 1) and speech language pathology (*n* = 1). These trainees were in master’s (*n* = 12), doctoral (*n* = 52) or postdoctoral (*n* = 6) training. Table 2 summarizes the overall characteristics of included papers along with trainee characteristics.

**Table 2:** Characteristics of included citations and trainees

Author(s), year	Country of origin	Study population	Study setting	Study design (reported and assessed by review team)	Trainee characteristics	
					Reported health/clinical discipline	Stage of training
Abdul Qader and King, 2015 [37]	Canada	Patients with ostomies	Qatar	Multi-methods approach (literature review + engagement)	Nursing	Master’s
Abelsohn et al., 2011 [38]	Canada	Graduate students at the University of Toronto, academics and community-based members of the lesbian, bisexual, trans and queer (LGBTQ) communities and organizations	Conference on LGBTQ health	Case study	Public health, nursing, health policy	Master’s and PhD
Allen and Hutchinson, 2009 [68]	Canada	Patients with end-stage renal disease	Hospital	Participatory action research using photovoice	Health science	Postdoctoral fellow
Atherton et al., 2016 [39]	Australia	Speech-language pathology graduates in Vietnam (graduates from the 2010–2012 Pha Ngoc Thach University of Medicine, speech-language training programme, in Ho Chi Minh City)	Vietnam	Participatory action research	Speech-language pathology	PhD
Baukus 2019 [69]	United States	14 to 15 refugee and immigrant teens	Community and university/college	Community-based participatory research, over a series of 8 after-school workshops	Public health	Master’s
Bellows Riecken, 2012 [89]	Canada	Individuals at risk for physical inactivity-young adults ranging from 18 to 35 who resided in Greater Victoria or Duncan, British Columbia (BC)	The first location provides distance education, job training certification, and alternative programmes for students completing lower grade level BC curricula The second location provides First Nations education programmes as well as high school equivalency classes for other students needing an alternative schedule or learning environment	Action research (Study 2)	Health education	PhD
Björnsdóttir and Svensdóttir, 2008 [105]	Iceland	Young Icelandic adults with learning disabilities who were born in the years 1974–1984 and are actively involved in various social activities such as self-advocacy, sports, religion and arts	Reykjavı´k, community	Life histories and follows an inclusive research paradigm, participatory action research	Social sciences	PhD
Bloodworth et al., 2004 [43]	United States	Graduate students	Three Chicago universities	Qualitative reflections on student experience with participatory community research	The majority of editors and contributors are academic faculty	Graduate students with different levels of training
Bishop et al., 2018 [97]	Canada	KT Canada Summer Institute trainees and early researchers involved in patient-oriented research	KT Canada Summer Institute (KTCSI)	NR	Nursing and medicine	NR
Burgess 2006 [44]	Canada	Primary healthcare nurse practitioners	Primary healthcare setting	Participatory action research study	Faculty of Human and Social Development, School of Nursing and Faculty of Education, Curriculum and Instruction	PhD
Burgess 2009[67]						
Boland, 2020 [100]	Canada	Paediatric clinical practice (clinicians, patients and parents)	Hospital	NR	Population health	PhD
Bowyer, 2018 [95]	Scotland	Health service users and healthcare providers of rural health services in Scotland	Scottish Highlands (an accessible rural community) and the western isles (a remote rural community)	Participatory action research using geographic information systems, mixed-methods research design (multiple-case study designs)	Health sector worker/dietitian	PhD
Donnelly, 2013 [56]	Canada	Four family health teams with occupational therapists	Family health teams in Ontario	Phase I used a multiple-case study design and phase II used a single-case design	Education	PhD
Cassidy, Burgess et al., 2019 [45]	Canada	Health System Impact Fellows—doctoral and postdoctoral trainees	NR	NR	Nursing	Postdoctoral fellow
Cassidy and Foley et al., 2019 [78]	Canada	Healthcare providers at medical, surgical and neuroscience unit	Hospital-based paediatric inpatient	Commentary about scoping review and stakeholder assessment of implementation barriers	Nursing	PhD
Campbell,2006 [93]	Canada	Members of the town, administration, an advocacy group and three focus groups, with one service provider group, one parent group and one youth group	Rural costal community on Prince Edward Island	Participatory action research + five stages of the Ottawa Model of Research Use (OMRU) knowledge translation framework	Nursing	PhD
Blum, 2006 [86]	United States	15 nurses	A hospital in South Florida	Participatory action research	Nursing	PhD
Conway et al., 2020 [81]	Canada	Healthcare providers	Urban hospital, Ireland	NR	Informational specialist	PhD
Dalal et al., 2009 [104]	United States	Physician-researcher postdoctoral fellows and the communities they worked in/ did their research with and community leaders	New Haven	Community-based participatory research	Medicine	Postdoctoral fellow
DeLemos et al., 2007 [47]	United States	People with chronic kidney disease in the Navajo population	Navajo community	Community-based participatory research	Engineering	PhD
Dewitt 2011 [103]	United States	Members of Oakland’s Promise Alliance	Oakland community	Participatory action research	Education	PhD
Dunn et al., 2020 [91]	United States	Eight students (youth, middle school boys)	Local middle school with a high percentage of students of low-income and minority backgrounds	A mixed-methods approach included a focus group and a pre-test/post-test with quantitative items and open-ended questions	Nursing	PhD
Feroz, 2009 [110]	United States	Early Head Start parents, Early Head Start practitioners, and community stakeholders in cycles of relevant dialogue during the development of a participatory action research project	University setting: Early Head Start programme located in rural western Pennsylvania	Qualitative inquiry using ethnography to observe a participatory action research	Department of sociology	PhD
Fletcher and Marchildon, 2014 [106]	Canada	Health system leaders at three levels: frontline units, senior leadership below the CEO level and senior executive leadership in the ministry and health regions	Saskatchewan, Canada health system	Participatory action research, Delphi method	Public policy	Postdoctoral fellow
Fletcher and Marchildon, 2018 [107]						
Gibbon, 2002 [40]	United Kingdom	Two voluntary sector organizations, focused on women	Kenya and Nepal	Qualitative reflective article	Community health	PhD
Gilhooly and Lynn, 2014 [41]	United States	Three Karen adolescent brothers	Two communities in Georgia, one in Iowa and one in Wisconsin	Collaborative ethnography	NR	PhD
Goessling and Doyle, 2009 [42]	Canada	Nine full-time students and active members of an at-risk student intervention programme	An urban high school in the Pacific Northwest	Participatory action research	Counselling psychology	Master’s
Smithers, Graeme and Mandawe, 2017 [46]	Canada	Urban First Nations men	Community	Community-based participatory health research project	Department of geography	PhD
González 2012 [71]	United States	Five co-researchers and five participants, all Latino immigrant parents of English language learner students	Northern California elementary school, located on the east side of San Jose in a predominantly Latino neighbourhood	Participatory action research	Education	PhD
Haywood et al., 2019[72]	United States	Adolescents and young adults with chronic conditions	Los Angeles County	Participatory action research	Occupational therapy	PhD
Hilario, 2018 [57]	Canada	Immigrant and refugee men living in Greater Vancouver, British Columbia	Community	Integrated knowledge translation using participatory video method	Nursing	PhD
Hohn, 1998 [65]	United States	The adult literacy programme participants, the student action health team and the public health and education agencies	Broad Street Learning Center in Lynn, Massachusetts. Lynn is known as "immigrant city", hosting many waves of immigrant groups since the turn of the century	Participatory action research with an empowerment focus	Education	PhD
IKTRN [98]	Canada	N/A	N/A	N/A This is an interview transcript	Nursing and medicine	Trainee A: PhD Trainee 2: medical student
Jervis, 2019 [59]	United Kingdom	Nursing staff, children and families visiting adult relatives in hospital	Large teaching hospital	Participatory action research	Nursing	PhD
Jull et al., 2019 [92]	Canada	Inuit community support workers (CSWs)	Ottawa, Ontario Nunavut in Inuit Nunangat, specifically the Qikiqtaaluk (Baffin) region	Qualitative study with two theory-driven phases: (1) Using consensus-building methods to tailor a previously developed shared decision-making (SDM) strategy with Inuit and developing training in that SDM strategy (2) Training CSWs in the SDM strategy, and then matching the CSWs with community member volunteers to test the SDM strategy	Occupational therapy	Postdoctoral fellow
Jones, 2012 [64]	Canada	51 students and 7 staff members from schools A and B	Uganda, school	Participatory action research	Global health	Master’s
Jones, 2019[62]	Australia	Aboriginal and Torres Strait Islander	Mayi Kuwayu: The National Study of Aboriginal and Torres Strait Islander Wellbeing	Strengths-based, mixed-methods approach (1) Cross-sectional analysis (2) Community-based participatory research (3) Descriptive epidemiology and an environmental investigation	Epidemiology and population health	Master’s
Kinman, 2017 [82]	United States	Paediatric residents rotating on the adolescent medicine rotation and the high school students enrolled in the women’s alliance elective class at the high school	School	Participatory action research	Education	Master’s
Khobzi and Flicker, 2010 [70]	Canada	The Positive Youth Project (Flicker): HIV-positive teens and young people The Trans PULSE Project (Khobzi): trans (transgender, transsexual and transitioned) people in Ontario	Ontario, Canada	Community-based participatory research (both) Positive youth project: qualitative interviews with HIV-positive youth Trans PULSE Project: in-depth quantitative survey	Health	PhD (both)
Lac and Fine, 2018 [74]	United States	High school students	School	Participatory action research	Education	PhD
Laur et al., 2020 [79]	Canada	Hospital units (directly related to patient nutrition)	Hospitals	NR	Public health and health systems	PhD
Leslie et al., 2010 [49]	United States	Hawaiian workers and administrators	4 health agencies, 3 social service agencies, and one faith-based organization	Community-based participatory research	Medicine	PhD
Lewis, 2020 [77]	Canada	Patients receiving implantable cardioverter-defibrillator	Canadian tertiary care centre	NR	Nursing	PhD
Lillehagen, 2017 [96]	Norway	Six physiotherapy institutes within primary healthcare. Clinical coordinators who were all experienced clinicians with more than 10 years of practice	Hospital, primary healthcare	Participatory action research	Medicine	PhD
Lind, 2006 [87]	Canada	Adolescents in North American society	School	Hermeneutically inspired, participatory action research	Nursing	PhD
Maher, 2018 [61]	Australia	Outbreak investigation: Members of the Aboriginal community, Yarrabah, Queensland, with confirmed or probable cases of mumps Evaluation and epidemiological study: Members of the Aboriginal community in Wadeye, Northern Territory	Outbreak investigation: Yarrabah, Queensland Evaluation and epidemiological study: Wadeye, Northern Territory	Study 1: secondary data analysis, mixed-methods approach Study 2: descriptive analysis Study 3: programme evaluation	Epidemiology and population health	Master’s
McCaig, 2019 [63]	Scotland	38 participants (18 patients, 14 staff, 4 carers, 2 students)	A 25-bed mixed-sex acute mental health ward	Appreciative action research	Mental health nursing	PhD
McHugh, 2008 [85]	Canada	Students (specifically young Aboriginal women, aged 14–18 years), teachers and staff of Nutana Collegiate	An urban high school (i.e., Nutana collegiate) in Saskatoon	Participatory action research	Kinesiology	PhD
Murdoch, 2006 [66]	Canada	Women with disabilities, an academic, a community leader of a local disability organization, a woman with disabilities and a graduate student in women’s studies	St. John’s, Newfoundland and surrounding areas	Feminist case study	Nursing	Master’s
Medcalf, 2008 [75]	Canada	Six elders, at least 70 years old	Community	Participatory action research	Education	PhD
Mitchell, 2018 [102]	United States	American Indian (AI) tribe located in the Midwest region of the United States	Small reservation community	Community-based participatory research using photovoice methodology	Institute for Policy and Social Research	PhD
Munro, 2018 [54]	Canada	Women who had caesarean section	British Columbia, hospital	NR	Interdisciplinary studies	PhD
Moll & Clements, 2008 [73]	Canada	Eight stakeholder groups; participants included a mix of representatives from human resource departments, occupational health, disability managers/employee assistance programme providers, managers or supervisors and consumers of mental health services Businesses represented included municipal service providers (e.g., police, transit commission), healthcare providers (large and mid-sized hospitals as well as community-based programmes), the educational system (school boards and postsecondary institutions), large and mid-sized retail and manufacturing businesses, and experts in the field who have addressed these issues in practice (e.g., disability managers, occupational psychiatrists, employees)	Research foundation	Multi-method, knowledge translation (reported as: an interactive process of KT)	Occupational therapy (Mental illness in the workplace)	PhD
Nadimpalli et al., 2016 [48]	United States	Sikh Asian Indian community	Community	Community-based participatory research	Nursing	PhD
Oosman, 2012 [83]	Canada	8- and 9-year-old children living in Île-à-la-Crosse, Saskatchewan Participants of focus group: eight individuals: non-Aboriginal and five Métis, four teachers, two healthcare professionals, one parent and one elder	Community	Participatory action research	Interdisciplinary studies	PhD
Ormel and Law, 2020 [53]	Canada	Cholera-affected populations in Haiti	Haiti	Participatory action research	Humanitarian	PhD
Pratt et al., 2019 [108]	Australia	Master’s in nursing students and registered nurse/ professor (a group of five academic staff, a mixture of sessional and permanent)	A metropolitan university in New South Wales, Australia	The co-construction processes with participatory practice development principles	Nursing	PhD
Ramage et al., 2020 [80]	Australia	People living with stroke	Stroke rehabilitation centre	Codesign method	Physiotherapy	PhD
Ramstetter, 2010 [88]	United States	174 students (kindergarten through grade 8)	Corryville Catholic school	Participatory action research	Health promotion and education	PhD
Reale, 2011[84]	United Kingdom	High school students, stakeholders and staff (administration to teachers)	Suburban comprehensive high school in southeastern Connecticut, USA	Whole-school, exploratory, single-case study, participatory action research	Education	PhD
Roberts and Jette, 2016 [109]	United States	Young women in the community (ages 11–17 years)	Community	Formative research, grounded in participatory research methodology	Public health	PhD
Robinson, 2007 [94]	Canada	Female adults (over the age of 21) who have, or have had, some form of mental illness (broadly defined) and who have participated in mental health-related research activities	US	Mixed-methods, cross-sectional survey approach	Social work	PhD
Sanderson et al., 2020 [99]	Canada	Postsecondary students with psychosis	Hospital and research setting	Mixed-methods systematic review	Nursing	Master’s
Schuch, 2017 [50]	United States	Hispanic immigrants in Charlotte Mecklenburg	Community	Community-based participatory research	Health	PhD
Shelton, 2012 [51]	United States	Upper elementary students	Elementary school	Participatory action research	Sociology	Master’s
Sim et al., 2019 [101]	Canada	Postdoctoral fellows (*n* = 46)	Fellowship launched in 2017–2018, through which postdoctoral fellows were colocated between a health system-related organization	Reflection paper	NR	Postdoctoral fellow
Suderman et al., 2020 [90]	Canada	Cancer survivors	Community	Multi-method IKT approach	Physical therapy	NR
van der Meulen, 2011 [76]	Canada	Canada’s oldest sex worker-run organization	Community	Action research methodology	Department of criminal justice and criminology, women studies	PhD
Videmšek and Fox, 2018 [55]	First author: Slovenia Second author: United Kingdom	First author: experiences of people with mental ill health living in group homes in Slovenia Second author: carers of people with schizophrenia	First author: Slovenia Second author: United Kingdom	First author: interpretive methodology adopting cooperative inquiry Second author: a participatory action research approach	Social work	PhD
Volpē, 2017 [60]	United States	High school students	Upward Bound programme located at a large public university in the Appalachian region of the United States	Participatory action research using photovoice	Education	PhD
Wilbricht, 2017 [52]	United States	American Indians and Alaska Natives	An Indian reservation in northern Arizona, and a rural, Indigenous community in western Alaska	Community-based participatory research	Communication	PhD
Wine et al., 2017 [58]	Canada	N/A	N/A	Scoping review	NR	Master’s

*NR* not reported, *N/A* not applicable

The included studies were mapped onto the KTA cycle [29] based on the reported research purpose and objectives, as follows: identify problem (*n* = 30) [37–67]; adapt knowledge to local context (*n* = 34) [37–40, 43, 44, 46, 49, 52, 55, 56, 61, 62, 64, 65, 67–86]; assess barriers/facilitators to knowledge use (*n* = 14) [39, 40, 52, 56, 63, 68, 72, 74, 81, 83, 87–90]; select, tailor, implement interventions (*n* = 11) [56, 63, 68, 80, 81, 83, 88–92]; monitor knowledge use (*n* = 25) [55, 63–65, 69, 73, 80, 83, 89, 91–107]; evaluate outcomes (*n* = 22) [48, 50, 51, 62, 64, 68, 71, 79, 83, 91, 93, 95, 100–110]; sustain knowledge use (*n* = 1) [79]. As shown, many studies comprised more than one KTA phase. However, some papers (*n* = 14) [40, 45, 46, 50, 51, 54, 70, 74, 80, 101, 104, 105, 108, 109] did not fit into the KTA phases, as they were more reflective in nature.

### Knowledge user engagement

Knowledge users were engaged in multiple stages of research: (1) research question (*n* = 42); (2) research proposal (*n* = 39); (3) administrative pre-launch (*n* = 30); (4) recruitment and data collection (*n* = 51); (5) data analysis (*n* = 45); (6) dissemination and implementation (*n* = 26). Seven studies did not report the stages of knowledge user engagement. We also recorded the level of knowledge user engagement based on the IAP2 Spectrum for Public Participation [111]: inform (*n* = 2); consult (*n* = 8); involve (*n* = 15); collaborate (*n* = 43); empower (*n* = 3). Four studies did not report the level of knowledge user engagement. See Table 3 for more detail.

**Table 3:** Characteristics of knowledge users and engagement

Author(s), year	Knowledge users	IAP2 level of engagement	Engagement stage CORE						Mode of engagement	Duration, frequency, timing
			1 Research question	2 Research proposal	3 Admin pre-launch	4 Recruitment and data collection	5 Data analysis	6 Dissemination and implementation		
			*N* = 42	*N* = 39	*N* = 30	*N* = 51	*N* = 45	*N* = 26		
Abdul Qader and King, 2015 [37]	Student’s academic supervisor, clinical experts (the physician lead for colorectal cancer services and two advanced clinical nurse specialists in the gastrointestinal programme), clinical pharmacologists	Involve					✓		Receiving advice and feedback (mode not described)	NR
Abelsohn et al., 2011[38]	LGBTQ members, students, faculty members	Involve		✓					Meeting, a callout for committee members sent through varies channels	NR
Allen and Hutchinson, 2009[68]	Patients with end-stage renal disease (ESRD), policy-makers, healthcare providers or anyone trying to improve the quality of life and treatment for patients living with ESRD, filmmakers working with patient populations	Collaborate	✓	✓		✓	✓	✓	Meeting and discussion	3 h
Atherton et al., 2016 [39]	Healthcare providers: new speech-language pathologists with undergraduate degrees in other health professions (e.g., physiotherapy, medicine, nursing)	Collaborate	✓	✓	✓	✓	✓	✓	Interview, meetings (e.g., Skype)	24–30 months
Baukus 2019 [69]	Independent community groups and a faculty member. Community partner is a local group of artists, designers, researchers, educators and social workers working together to improve access to quality mental healthcare services	Collaborate	✓	✓					Meetings and conference calls	NR
Bellows Riecken, 2012 [89]	Community partner organization	Collaborate				✓	✓	✓	Survey	NR
Blum, 2006 [86]	Nurse preceptors, clinical faculty, nurse preceptors, hospital nurse educators and nursing administrators	NR			✓	✓			Action plan	13 months, from May 2005 to June 2006
Björnsdóttir and Svensdóttir, 2008 [105]	Young Icelandic adult who is a self-advocate with learning disabilities	Collaborate						✓	NR	Once per week for 6 months
Bloodworth et al., 2004 [43]	Community researchers	NR	NR						NR	NR
Burgess 2006 [44]	Nurse Practitioners	Involve				✓	✓		Meeting, written handout, discussion	A total of five meetings, 2–3 h in length
Burgess 2009 [67]										
Boland, 2020 [100]	Clinician-scientist (medical director of the Children's Hospital of Eastern Ontario [CHEO] shared decision-making programme, paediatric endocrinologist), registered nurse	Involve	✓	✓	✓	✓	✓	✓	Meeting, and regular team meetings, regular process updates	Regular team meetings (3–4 per year)
Bowyer, 2018 [95]	Professionals engaged in health planning within NHS Scotland, community stakeholder groups : – Remote and rural area, 7 community groups including community council, primary school parent group, retrained fire service, patient participation group, community shop committee, community hall committee – Accessible rural community, 5 community groups including primary school parent groups, friendship group, mother and toddler group, community council, patient participation group	Collaborate				✓	✓	✓	Focus group, participatory mapping techniques, interviews	NR
Bishop et al., 2018 [97]	A total of 15 faculty and facilitators, including two patient advisors. The two patient advisors represented independent patient advisory networks and were active in their respective provincial Strategy for Patient-Oriented Research (SPOR) units	Involve				✓			NR	NR
Campbell, 2006 [93]	Paediatric community in Prince Edward Island, Canada	Consult	✓			✓			Group meetings, group interviews	NR
Cassidy, Burgess et al., 2019 [45]	A group of Health System Impact (HSI) postdoctoral fellows	Collaborate	✓	✓	✓	✓	✓		NR	NR
Cassidy and Foley et al., 2019 [78]	Manager of the medical, surgical and neuroscience unit, clinical nurse leaders, researchers, administrators	Collaborate	✓	✓	✓	✓	✓		KT research processes	NR
Conway et al., 2020 [81]	Healthcare providers working in the neonatal and obstetric departments. Five knowledge users (nurses and midwives working in the neonatal and obstetric departments) made up an on-site implementation team	Collaborate	✓	✓				✓	Reaching out to key informants within the targeted hospital departments and through word-of-mouth recommendations, focus group and interview	Six group sessions, 9 months
Dunn et al., 2020 [91]	Administrator, education consultant, two nonviolence educators, teacher, nursing faculty member and a doctoral student who possessed paediatric expertise	Collaborate						✓	A series of discussions, evaluation included a pre-/post-test, open-ended written questions, a focus group	NR
Dalal et al., 2009 [104]	Steering community consisting of community leaders and community-focused academicians	Involve	✓	✓	✓	✓	✓		Interview, seminar, structured feedback, meeting, retreat	NR
DeLemos et al., 2007 [47]	Navajo people	Collaborate	✓	✓		✓			Cultural education	NR
Dewitt, 2011 [103]	Policy-maker, America’s Promise Alliance members, Oakland’s Promise Alliance members	Collaborate				✓	✓		Dialogue, brainstorming, critical reflection, group meetings, field notes from formal and informal meetings, observation, and community consultation, interview	Two 2-h meetings
Donnelly, 2013 [56]	Occupational therapists, the executive director and the lead physician at the family health team, membership of the evaluation committee (all members of the memory clinic, one Alzheimer Society member, the executive director)	Collaborate	NR						Informal meeting, case study, evaluation committee was formed, member-checking	NR
Fletcher and Marchildon, 2014 [106]	Each round of the study included up to four health system actors as both research partners and participants Participants in the study represented three levels of authority within the health system and were categorized into three corresponding participant groups: – Group 1 included governance and directional leadership at the ministry and health region executive level – Group 2 consisted of shared services senior leadership immediately below CEO level at collective and individual project stream levels – Group 3 included frontline leadership of existing business units within each project	Collaborate	✓	✓	✓	✓		✓	Delphi technique	NR
Fletcher and Marchildon, 2018 [107]										
Feroz, 2009 [110]	Early Head Start parents, Early Head Start practitioners, and community stakeholders	Collaborate	✓				✓	✓	Group sessions, focus groups	Weekly, 22 group sessions
Gibbon, 2002 [40]	Academics/researchers	NR	NR						NR	NR
Gilhooly and Lynn, 2014 [41]	Three adolescent Sgaw Karen brothers, and Karen communities	Collaborate	✓	✓	✓	✓	✓		Semi-structured interviews, informal meeting	40 interviews, biweekly
Goessling and Doyle, 2009 [42]	High school students, teachers, counsellors, therapists, mentors and others who desire to have positive relationships with teenagers	Involve	✓	✓	✓	✓	✓	✓	Tutoring, mentoring, leadership development, summer enrichment programmes, photo exhibition	20 weeks
González, 2012 [71]	Latino immigrant parents of English language learner students	Collaborate				✓	✓	✓	Meeting, focus group	NR
Smithers, Graeme and Mandawe, 2017 [46]	Indigenous person research assistant, urban First Nations men	NR	✓	✓	✓	✓	✓		NR	NR
Haywood et al., 2019 [72]	Adolescents and young adults with chronic conditions, researchers, healthcare providers and related stakeholders	Collaborate	✓	✓				✓	Virtual or in-person meetings, phone or email communication, conference, academic meetings	At least monthly
Hilario, 2018 [57]	Six immigrant and refugee young men (self-identified as an immigrant and refugee young men from China, Philippines, Mexico, Colombia and Afghanistan), an advisory group comprising service providers and programme leaders	Collaborate	✓			✓	✓	✓	Conversations, structured meetings with advisory group, unstructured consultations, dialogue	NR
Hohn, 1998 [65]	Student action health team, literacy programme environment administration and staff, external empowerment public health and education agencies- funders, administration and staff	Collaborate	✓				✓	✓	Meeting, field notes, written communications, informal conversations, interviews and semi-structured interviews, discussions, surveys	Two surveys, two group interviews, eight individual interviews
IKTRN [98]	Trainee A: clinical team of a paediatric inpatient unit Trainee B: a team of people from Island Health, organized speakers from Island Health, community organizations, and members from the Nanaimo municipality	Consult			✓		✓		Trainee A: NR Trainee B: written report	NR
Jull et al., 2019 [92]	A steering committee that consisted of members from Inuit-led or Inuit-specific (or both) health and related organizations that support Inuit in cancer care systems	Consult	✓			✓	✓	✓	Consensus-building methods, feedback	NR
Jervis, 2019 [59]	The Medicines for Children Research Network (MCRN), Young Persons Advisory Groups (YPAGs), National Institute for Health Research network -The YPAGs consist of 10 to 15 members who are aged 8–19 years -The groups consisted of a total of 23 young people aged 8–17 years comprising 16 girls and 7 boys -Nursing staff	Consult and Involve	✓	✓	✓	✓	✓		Focus group, face-to-face discussion	NR
Jones, 2012 [64]	Students and academic staff from the selected schools	Involve	✓	✓	✓	✓	✓	✓	Semi-structured focus groups and interviews	NR
Jones, 2019 [62]	Aboriginal and Torres Strait Islander: Palngun Wurnangat Aboriginal Corporation (PWAC) and Kardu Lurruth Ngala Purrungime (KLNP) committee	Consult	✓			✓	✓		NR	NR
Khobzi and Flicker, 2010 [70]	The Positive Youth Project (Flicker): service providers, unspecified "partnering organisations" and HIV-positive teens and young adults, a public health research unit, a hospital, a national AIDS service organization, grassroots POZ youth group The Trans PULSE Project (Khobzi): trans people, representatives from trans community organizations, academic partners, and unaffiliated trans community members, a primary healthcare centre, a community centre	Collaborate	✓	✓	✓	✓	✓	✓	Meetings, interview	35 interviews
Kinman, 2017 [82]	University of California, San Francisco Fresno Pediatric Residency Program and the women’s alliance class at the high school during the school years 2015–2016 and 2016–2017	Collaborate	✓	✓	✓	✓	✓	✓	Discussion, survey,	NR
Lac and Fine, 2018 [74]	The individual youth researchers in our cohort of 11 (high school students)	Collaborate	NR						Dialogical approach	NR
Laur et al., 2020 [79]	Local champions, who were already (in most cases) employed as dieticians within managerial or leadership roles	Empower	✓	✓	✓	✓	✓		Monthly group calls	Monthly
Leslie et al., 2010 [49]	10 Native Hawaiian-serving organization workers, administrators, advisors from the worksites	Involve	✓	✓	✓	✓	✓		Meetings, interviews, focus groups for open discussion	9 focus groups, 90 min
Lewis, 2020 [77]	Steering committee: multidisciplinary team of knowledge users (director of the arrhythmia service, the ambulatory device clinic nursing manager, a device clinic registered nurse, expert researchers in patient decision aids development, two patients who had previously undergone implantable cardioverter-defibrillator replacement and the spouse of a patient with an implantable cardioverter-defibrillator	Involve		✓			✓		Steering committee, email, telephone, meeting, feedback	Steering committee met twice
Lillehagen, 2017 [96]	Researchers, physiotherapist, physiologist, clinical coordinators	Collaborate	✓	✓		✓			Meetings with structured agenda	NR
Lind, 2006 [87]	Adolescents	Collaborate	✓	✓	✓	✓	✓		Interviews	NR
Maher, 2018 [61]	Aboriginal health workers, locally identified Aboriginal community researchers, including members of the Kardu Lurruth Ngala Purrungime committee, department of the Prime Minister and Cabinet Indigenous Affairs Group Policy Analysis and Evaluation Branch	Collaborate		✓	✓				Survey, training	NR
McCaig, 2019 [63]	Patients, staff, carers and students, mental health institution staff, policy-makers, educationalists and researchers	Involve				✓	✓	✓	Interviews, focus groups and reflection groups	NR
McHugh, 2008 [85]	The known sponsor (the director of Nutana’s Integrated School-Linked Services programme) and a group of 7 young Aboriginal women who represented the Core Group. Students, teachers, and staff at Nutana Collegiate, and also a known sponsor who was embedded in the administration	Empower	NR						Interviews, focus groups	NR
Medcalf, 2008 [75]	Six elders with a considerable diversity, including age, gender, socioeconomic status, formal education, religious affiliation, former occupation, current household and type of housing, sexual orientation, marital status, whether they have children or grandchildren, their interests as well as their life experience in general. All are white, speak English as their first language and all but one has grown up in southern Ontario	Collaborate	✓	✓	✓				One-page autobiography to present to the group, workshop, individual conversations	NR
Mitchell, 2018 [102]	American Indian tribe	Involve				✓	✓	✓	Recruitment flyer, attendance of local events including annual gatherings and reoccurring activities	NR
Moll and Clements, 2008 [73]	Employees with mental health issues, insurers, healthcare professionals, human resource personnel, researchers and education, unions, managers, occupational health and disability management providers	Collaborate				✓	✓		Focus group, feedback, meetings	NR
Murdoch, 2006 [66]	Women with disabilities, an academic, a community leader of a local disability organization, a graduate student in women’s studies, government, researchers, policy-makers	Collaborate	✓			✓			Email communication, interview, personal letter	NR
Munro, 2018 [54]	Patients, providers, and policy-makers in British Columbia	Collaborate	✓	✓	✓	✓	✓		Ongoing feedback, co-writing	NR
Nadimpalli et al., 2016 [48]	Sikh Asian Indian community partners (including social service agencies, advocacy groups, and healthcare providers) serving the South Asian community)	Collaborate	✓	✓	✓	✓	✓		Coalition meetings, relationship-building activities	2 years
Ormel and Law, 2020 [53]	National and international staff member from the nongovernmental organization (NGO) Action Contre la Faim, different governmental agencies, NGOs, traditional healers	Inform and Collaborate	✓	✓		✓	✓		Telephone meeting, email communication, focus group discussion	NR
Oosman, 2012 [83]	Meti's Community	Collaborate	✓	✓	✓	✓	✓	✓	Phone discussion, verbal agreement, face-to-face meetings	NR
Pratt et al., 2019[108]	Masters in nursing students	Involve	✓	✓	✓	✓	✓		Two exercises to clarify values and ways of working, meetings, follow-up feedback session	NR
Ramage et al., 2020 [80]	The research team: four knowledge-user partners (one person with a lived experience of stroke, two physiotherapists with research experience, one exercise scientist experienced in telehealth exercise with stroke survivors) and five researchers (one PhD candidate [physiotherapist] and four PhD supervisors with research expertise in physiotherapy and nutrition and dietetics) Knowledge-user informants: healthcare workers, such as doctors, nurses, physiotherapists, managers; stroke survivors; carers; behaviour change researcher	Collaborate	✓	✓	✓	✓			Workshops, individual interviews, teleconferencing into workshops	NR
Ramstetter, 2010 [88]	Students, teachers, staff, parents, volunteers and the school board	Collaborate	✓	✓	✓	✓	✓	✓	Meeting, written communication	NR
Reale, 2011 [84]	Students and staff at the high school	Consult				✓		✓	NR	NR
Roberts et al., 2016	Guardians of the youth, urban Indian young women	Collaborate				✓	✓		Semi-structured interviews, art project	
Robinson, 2007[94]	The four individuals served as consultants in mental health-related research and had self-disclosed that they had (or previously had) a mental illness	Consult		✓			✓		Email communication, telephone communication, formal letter, written communication including journaling, feedback (via email)	NR
Sanderson et al., 2020 [99]	One registered nurse in a psychiatric outpatient setting, two medical doctors and one social worker in a postsecondary (PS) institution, one nursing student interested in mental health, one student with lived experience of psychosis, and one director of health and wellness services in a PS institution, one occupational therapist working for a first-episode psychosis programme, a nursing professor of mental health at a PS institution, a mental health policy advisor at a PS institution, and a research scientist involved with improving youth mental health services and supports	Collaborate	✓	✓	✓	✓	✓	✓	Introductory meeting, written partnership agreement, advisory panel, meetings, email or telephone communication, presentation	NR
Schuch, 2017 [50]	Partners included health and social service providers, educators, Hispanic foreign-born residents and a community advisory board, which included representatives from the local school system, the county health department, and the city of Charlotte	Collaborate	NR						Key informant interviews, focus groups, a photovoice project, and community forums	NR
Shelton, 2012 [51]	First project: five graduate students and environmental sociology professor, a group of fourth-, fifth- and sixth-grade students at an upper-elementary school in Hammond, Louisiana Second project: 12 graduate students and professor, fourth-, fifth- and sixth-grade students at the same upper-elementary school in Hammond	Collaborate				✓	✓		Survey, meeting, dialogue, journal, discussion	NR
Sim et al., 2019 [101]	Postdoctoral Fellows	Collaborate	✓	✓	✓	✓			NR	NR
Suderman et al., 2020 [90]	Cancer survivors and caregivers	Inform				✓			Questionnaires, semi-structured interviews	NR
van der Meulen, 2011 [76]	Sex workers	Collaborate	✓	✓		✓		✓	Informal meetings, email conversation, interview	NR
Volpē, 2017 [60]	Young people in a rural Appalachian community	Involve				✓	✓		Class format, photographs, discussion	In class, once per week
Videmšek and Fox, 2018 [55]	First author: five people with personal experience of mental ill health Second author: a steering group of stakeholders including people from professional, family-caring and research backgrounds	Collaborate	NR						NR	NR
Wilbricht, 2017 [52]	Primary community partners (the general managers of two tribal radio stations), community members	Empower	✓	✓	✓	✓	✓		Written agreement, interviews	NR
Wine et al., 2017 [58]	Health, and knowledge translation experts	Consult	✓	✓	✓	✓	✓		NR	NR

### IKT strategies

Overall, there was a lack of reporting on the use of IKT strategies. As detailed in Table 3, meetings were the most common mode of engagement with knowledge users (*n* = 27). Written communication, such as handouts or letters, was also used (*n* = 11), as was email (*n* = 7) and phone communication (*n* = 6).

### Barriers and facilitators to the IKT approach

Table 4 summarizes the key barrier themes identified from the included papers. Trainees’ lack of knowledge and skills posed challenges for IKT research [48, 71, 72, 74, 79, 84, 97, 109]. IKT knowledge and skills were related to knowing how to apply an IKT approach and specific procedural techniques related to IKT research processes. Also, trainees reported that their “outsider” status from the health system made the knowledge user engagement challenging [44, 51, 52, 67]. In terms of the IKT process, trainees reported that competing priorities among trainees (e.g., coursework, thesis/project completion), knowledge users (e.g., patient care, service delivery, resource use) and university institutions (e.g., timely thesis completion, resource use) posed challenges to the IKT approach [62, 65, 70, 72, 78, 79, 94]. Furthermore, knowledge users can have full schedules, making it difficult to find a common time to meet as a team [47, 89]. It was also difficult for trainees to define the scope of the research project that fulfilled knowledge user needs, aligned with health system priorities, and adhered to institutional guidelines (i.e., thesis requirements) [44, 63, 67, 74, 83]. The time-limited nature of graduate programmes also posed challenges to trainees [64, 81, 99]. Trainees also experienced power dynamics within the academic and health system institutions, which further contributed to the difficulties of knowledge user engagement [44, 51, 56, 72, 93]. Lastly, it was challenging to meet knowledge users in person when they lived too far and had to travel a long distance [39, 47, 52].

**Table 4:** Trainee-reported barriers to IKT

COM-B [147] component	TDF Version 2 [148] 14 Domains (definition)	Themes	Levels
Capability	Knowledge (An awareness of the existence of something)	Lack of knowledge and skills Know-how Procedural techniques (related to research processes) Interpersonal skills related to relationships	Individual
	Skills (An ability or proficiency acquired through practice)		
Motivation	Social/professional role and identity (A coherent set of behaviours and displayed personal qualities of an individual in a social or work setting)	Trainee as an outsider Shifting between community, health system, academia Trainees do not have insider privilege within any health organizations Trainees’ non–Native person status doing research in partnership with Indigenous communities	Individual, interpersonal
	Goals (Mental representations of outcomes or end states that an individual wants to achieve)	Competing priorities of knowledge users, trainees and university Containing scope for thesis project University guidelines and expectations Health system priorities	Interpersonal, organizational
Opportunity	Social influences (Those interpersonal processes that can cause individuals to change their thoughts, feelings or behaviours)	Power dynamics Power/pressure from institutions Administrative pressure Intergroup relationships or group dynamics Connecting, communicating and building relationship Push-back and scepticism	Interpersonal, organizational
	Environmental context and resources (Any circumstance of a person’s situation or environment that discourages or encourages the development of skills and abilities, independence, social competence and adaptive behaviour)	Lack of funding to support knowledge user participation Barriers to engagement due to physical location/distance Lack of time for trainees Engagement activities Trainee’s institutional programme Institutional restrictions University thesis guidelines Research ethics board Health organizations	Organizational

Table 5 summarizes the key facilitator themes identified from the included papers. Some trainees gained the necessary knowledge and skills to undertake IKT research in specialized graduate courses focused on applied health research [69, 82]. Similarly, trainees viewed themselves as facilitators [39, 55, 65, 110] and reported that facilitation skills helped with knowledge user engagement [38, 53, 65, 80, 91, 102]. For example, trainees aimed to use clear and common language to facilitate knowledge user engagement activities [80]. Additionally, trainees’ flexibility and problem-solving skills were helpful when engagement challenges required quick adaptations [45, 53, 91]. Trainees also reported that being an “insider” was a facilitating factor for doing IKT research [63, 76, 88, 110]. A pre-existing relationship with knowledge users or previous experience in the related field helped with building partnerships [70, 76]. Trainees also reported that trusting relationships and a safe place for partnership development were facilitating factors for knowledge user engagement [38, 50, 65, 66, 72, 80, 83]. This includes promoting a team culture that respects diverse perspectives. Trainees described using agendas and written agreements, such as a memorandum of understanding, to help set common goals early in the IKT process [65, 83, 98]. Supervisors played an important role in facilitating trainees’ IKT involvement [69, 70, 80, 100]. Trainees reported that having supervisors or committee members with expertise in IKT was helpful to guide them through the research partnership.

**Table 5:** Trainee-reported facilitators of IKT

COM-B component	TDF Version 2 [148] 14 Domains (definition)	Themes	Level
Capability	Knowledge (An awareness of the existence of something)	Education/coursework on research partnerships	Individual
	Skills (An ability or proficiency acquired through practice)	Facilitation skills Use of clear and common language Trainee’s reactivity and problem-solving skills	
	Behavioural regulation (Anything aimed at managing or changing objectively observed or measured actions)	Trainee’s flexibility and reflexivity Adapting expectations Project adaptability Fluidity between knowledge user and academia Self-reflection	
Motivation	Social/professional role and identity (A coherent set of behaviours and displayed personal qualities of an individual in a social or work setting)	Trainee as an insider Pre-existing relationship with knowledge user Previous experience in the field (e.g., clinical background) Ongoing efforts to maintain meaningful relationship Trainees view themselves as a “facilitator”	Individual, interpersonal
	Goals (Mental representations of outcomes or end states that an individual wants to achieve)	Setting common goals early when doing research and setting goals in knowledge user meetings Clear plan Memorandum of understanding (MOU) Planned agendas	
Opportunity	Environmental context and resources (Any circumstance of a person’s situation or environment that discourages or encourages the development of skills and abilities, independence, social competence and adaptive behaviour)	Financial resources Academic resources Time/scheduling to support partnership Person and environment interaction Straddle both environments (i.e., community, institution, hospital)	Interpersonal, organizational
	Social influences (Those interpersonal processes that can cause individuals to change their thoughts, feelings or behaviours)	Trusting relationship and creating safe place for partnership Team transparency Respect for diverse perspective Existing relationship and/or partnership Supportive group dynamics Knowledge users who support the research Partners’ aligned interest with the research IKT expert support Supervisors and committee members	Interpersonal, organizational

**Fig. 2:**
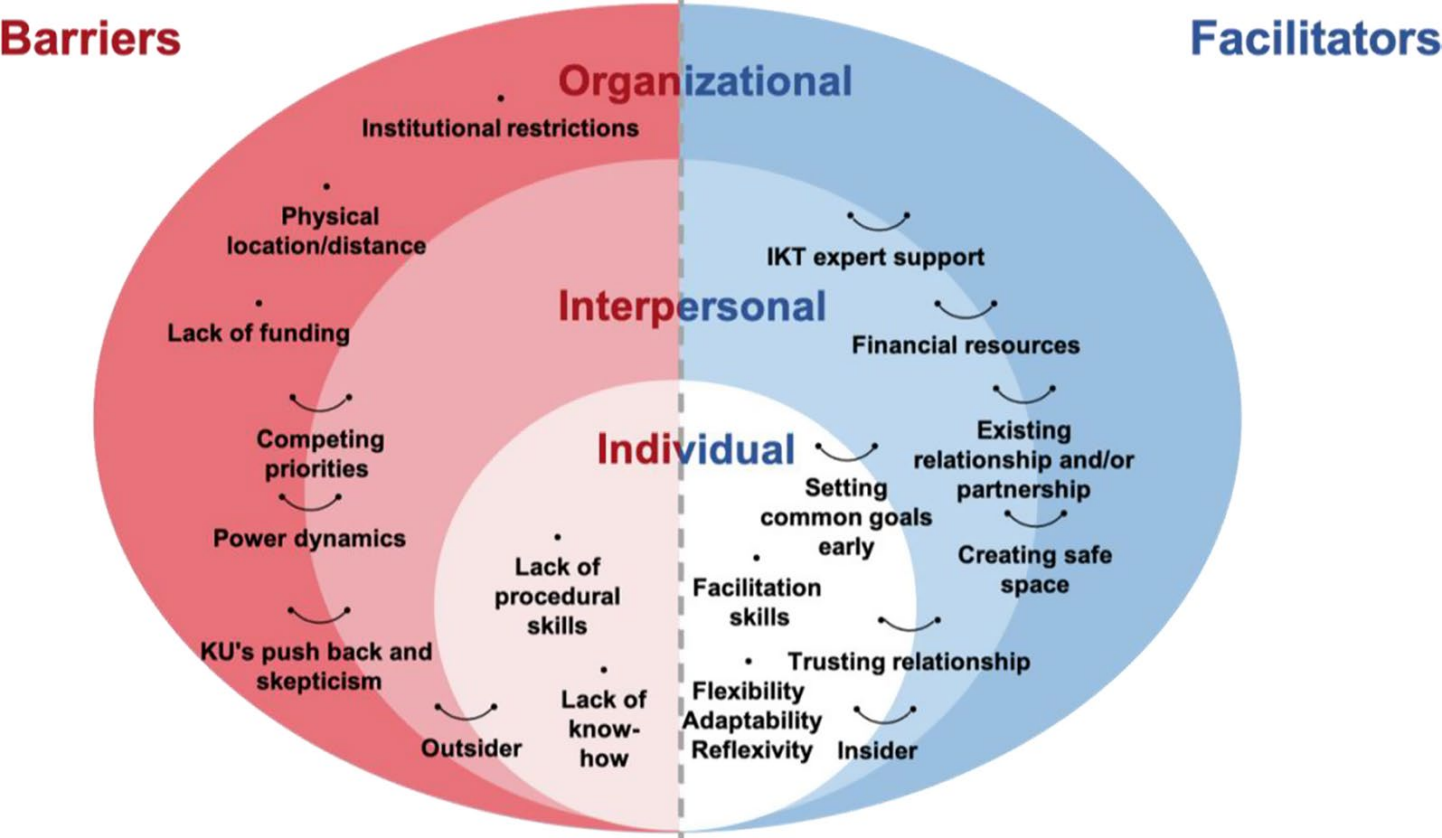
Barriers and facilitators to IKT/research partnerships in trainee-led research

Figure 2 represents a modified illustration of McLeroy’s social-ecological model [35] to help visualize where the reported barriers and facilitators exist within the trainee’s research ecosystem (i.e., individual, interpersonal and organizational levels). As shown in Tables 4 and 5, barriers and facilitators have been categorized to corresponding level(s) to guide interpretation of the data. First, the individual level represents trainees themselves. Second, the interpersonal level represents people that trainees interact with, such as supervisors and knowledge users. Lastly, the organizational level represents institutions, including universities and health systems, as well as communities. Identified barriers and facilitators are not static or absolute; as represented with connected lines, some of these barriers and facilitators exist across the three levels. Further, the three levels demonstrate the complex nature of individual trainees within a broad micro-, meso-, macro-system. Some of the barriers are the direct opposite of facilitators; for example, lack of funding and financial resources are reciprocal barriers and facilitators.

### IKT impact and outcomes

As shown in Table 6, reported IKT impact and outcomes were related to immediate (*n* = 38), intermediate (*n* = 40) and/or long-term (*n* = 15) outcomes of partnership formation. Twenty studies did not report outcomes related to IKT or other research partnerships. See Table 6 for more detail.

**Table 6:** Reported outcomes

Outcomes	Frequency (*N*)	References	Examples from included papers
*Immediate outcomes of partnership formation*	Total = 38		
Value for different perspectives/broadened perspective	*N* = 13	[37, 38, 46, 57, 61, 75, 76, 78, 80, 84, 96, 97, 106]	Bringing in diverse perspectives from different stakeholder groups
Mutual understanding of language, work style, needs and constraints	*N* = 12	[46, 47, 54, 62, 74, 77, 79, 81, 88, 94, 97, 103]	Inclusion of strategies to support a use of common language during workshops
Appreciation for collaborative process	*N* = 8	[38, 42, 47, 48, 50, 68, 69, 105]	Trainees reported that they experienced the value of collaborations
Capacity developed by researchers and decision-makers	*N* = 5	[37, 69, 72, 98, 109]	Trainees and/or partners gained experience (e.g., public speaking, professional networking and writing)
*Intermediate outcomes of partnership formation*	Total = 40		
Enhanced relevance of research	*N* = 18	[37, 47, 48, 63, 72, 73, 76, 77, 80, 84, 90, 97–101, 103, 106]	Trainees reported improved relevance of the research being conducted and/or its findings
Awareness and acceptance of research	*N* = 11	[42, 45, 52, 71, 76–80, 85, 95]	Gaining approval or “buy-in” of research from partners
Conduct of research	*N* = 5	[38, 46, 58, 91, 109]	Trainees reported that partnership approaches allowed for an improved research process
Identification of research questions	*N* = 3	[39, 94, 96]	Developing research questions together
Research output	*N* = 3	[50, 70, 72]	Co-authorship publication with knowledge users
*Long-term outcomes of partnership formation*	Total = 15		
Use of research in practice/policy	*N* = 9	[67, 76, 80, 82, 83, 95, 101, 106, 110]	Development of relevant practice change(s) in hospital or community
Scale-up/spread of research	*N* = 5	[37, 38, 41, 88, 100]	Research is spread to new areas
System/health service outcomes	*N* = 1	[77]	Changes in clinic processes and increased clinicians self-efficacy

Not reported *N* = 20

## Discussion

This scoping review describes how IKT or other research partnership approaches have been applied in thesis and/or postdoctoral health research. We identified 74 papers from published and grey literature sources to be included in the review. Overall, study findings provide insights for trainees interested in IKT or other research partnership approaches, and offer guidance on how to apply an IKT approach to their research. Further, the review highlights the important role that academic supervisors, knowledge users, and academic and health system institutions play in providing support and infrastructure to facilitate IKT or other research partnerships in trainee-led health research.

### IKT/research partnership principles, strategies and/or tools

The majority of studies involved collaboration with knowledge users in the research question development, recruitment and data collection stages of the research process. We used the IAP2 Spectrum  [30] as a way to categorize the levels of knowledge user engagement. It is important to note that, as reviewers, we had to make inferences on the level of engagement due to a lack of reporting on IKT and research partnership principles and strategies. We categorized the majority of studies (*n* = 43) in the collaborate stage, which indicates a high level of partnership in the decision-making process. This is not surprising, as IKT and research partnerships are rooted in deliberate partnership and shared decision-making throughout the research partnership from start to finish [112]. There are distinct differences between research partnerships that empower partners to be active participants in the shared decision-making process and lesser levels of engagement, such as communication, information-sharing and consultation [113]. The included papers described the former, where trainees made deliberate efforts to support research collaboration with knowledge users and described this partnership in detail. While the IAP2 Spectrum  was a useful framework for categorizing engagement, its origins are in broad public participation [111], and it does provide a comprehensive representation of the types of knowledge users involved in health research (i.e., patient, caregivers, healthcare providers, health system leadership and policy). Efforts are needed to develop a clearer set of IKT principles and strategies for knowledge user engagement in the health context.

### Barriers and facilitators to using IKT or other research partnership approaches in trainee-led health research

This review illustrates the intersecting barriers and facilitators for IKT and research partnership approaches at the individual, interpersonal and organizational levels (Fig. 2). Many of the reported facilitators were reciprocal to the barriers and have the potential to support IKT or research partnerships, including partnership skill development, co-creation of common goals and leveraging research programme partnerships. We used the COM-B model of behaviour [33] to categorize reported barriers and facilitators related to trainees’ capability, opportunity and motivation for using an IKT or research partnership approach.

#### ﻿Capability

Our review identified several capability-related barriers and facilitators at the individual trainee level for engaging in IKT research, including IKT knowledge and skills, and flexibility in the research approach. These findings align with recent research highlighting the lack of researcher preparation in research partnership approaches [11, 114]. Building on this known gap in the literature, our review offers a clearer understanding of what types of knowledge and skills are needed to support researcher preparation for collaborative health research, including the development of facilitation skills. Facilitation is defined as “the process of enabling (making easier) the implementation of evidence into practice” [115]. It supports a purposeful two-way process of change that focuses on building trusting relationships and establishing common goals between the facilitator and those engaged in making the change [116, 117]. More formal training efforts are needed to support facilitation skill development for health research trainees. Dogherty and colleagues developed a taxonomy of 53 facilitation strategies, ranging from providing assistance with certain tasks, to a more holistic process that empowers change in individuals’ attitudes and ways of thinking and working. This taxonomy may be a helpful starting point to develop trainees’ skills as facilitators in the research process, which to date has not been a primary focus in traditional academic programmes.

Previous research has identified core competencies that trainees need for applied health research and knowledge translation [118, 119]. A baseline self-assessment on these competencies may be helpful for trainees interested in developing their IKT or other research partnership skills [120]. Further, several training programmes exist to provide trainees with experiential learning opportunities in IKT or other research partnerships. For example, the Canadian Institutes of Health Research (CIHR) Health System Impact (HSI) Fellowship was designed to modernize doctoral and postdoctoral training to better equip researchers with the professional and research skills needed to address complex health system challenges [121]. HSI doctoral and postdoctoral research fellows are embedded in health system organizations to develop Canadian Health Services and Policy Research Alliance’s 9 enriched core competencies, understand the intricacies of health system delivery, and partner with members of the health system to support relevant research efforts [121]. Similarly, in the United States, AcademyHealth developed the Delivery System Science Fellowship to provide experiential learning and professional development opportunities for postdoctoral trainees [122]. These types of training programmes offer formalized experiences focused on development of professional skills not currently emphasized in health services graduate training. Trainees may wish to consider these types of formalized training programmes to further their IKT skills development and better support the use of a research partnership approach in their graduate or postdoctoral research.

#### ﻿Opportunity and motivation

Successful research partnerships require more than individual trainees having the right skills and willingness to engage with knowledge users. The majority of barriers and facilitators identified in this review were categorized in the interpersonal and organizational levels, specific to intersecting opportunity and motivation determinants that either support or hinder research partnerships for trainees.

Trainees reported that their status as an insider or outsider to the organization/community was a key facilitator or barrier for building partnerships. This is consistent with Dwyer and Buckle’s [123] previous work; having an insider membership typically gives an advantage to researchers, as it creates a sense of trust and openness from the participants. However, researchers are never completely insiders or outsiders, and their status does not make for a better or worse researcher [123–125]. Dwyer and Buckle [123] challenge the dichotomous insider and outsider identity of researchers and propose that “space between” is critical. In this “space between,” [123], researcher’s positionality is complex and fluid in nature [126] and can impact the research topic, epistemology, ontology and methodology [127]. Therefore, when trainees engage in IKT, it is more important that they practice self-reflexivity to understand their positionality in the study rather than focus on whether they are insiders or outsiders. Moreover, this is consistent with our findings on trainees reporting reflexivity as a facilitator for engaging in IKT research. We encourage trainees to reflect on the commonalities and differences they share with their research partners and how their positionality in the "space between" might affect research relationships. From there, trainees may have a better sense of how to navigate relationship-building with different knowledge user groups.

Academic supervisors play an important role in supporting trainees to navigate the “space between” and develop effective research partnerships with knowledge users. Our findings reinforce previous research on leveraging pre-existing research relationships to support IKT [18, 128] and highlight the value of research programme partnerships to facilitate trainee IKT research. Research programme partnerships and longer-term relationships, as opposed to one-off project partnerships, offer time and space for clear articulation of expectations, which allows for effective collaboration and mutual gain [129]. If research programme partnerships exist, it is easier for trainees to align their project within their supervisors’ larger research programme, which includes clearly developed practice linkages and existing research partnership supports [128]. Further, research programme partnerships ensure continuity of relationships between academia and the health system; when a trainee finishes their training and moves on to their next career stage, the supervisor can maintain the research partnership with the knowledge user. There may also be instances where trainees bring their own knowledge user partnerships into their academic training. For example, several papers described an existing role that trainees had within a community or organization, in which they used their partnerships to build a research study [76, 88]. In this scenario, supervisors should respect this pre-existing relationship and offer support on the academic research process to further develop the existing relationship into an effective research partnership.

Our findings also highlight how interpersonal influences can help and/or hinder the research partnership. The interpersonal and group dynamic barriers and facilitators to research partnerships illustrate the inherent relationship focus of IKT. Previous reviews of IKT-related literature have highlighted similar relationship-focused barriers and enablers to partnership research [4, 5, 17, 130]. Power dynamics are often at the core of research partnerships [131]. Several included studies highlight the influence of power hierarchies on the trainee experience with IKT, including pressure from both the academic and health institution to meet certain deliverables and timelines. This is particularly relevant for trainees who are already challenged with power hierarchies within the academy and may be entering into additional power differentials with knowledge users. Establishing mutually respectful relationships requires trust-building and being trustworthy to promote power-sharing and co-create knowledge [130]. Humility is an emerging area of interest in the field of IKT for developing meaningful and trustworthy research partnerships [132]. Humility stresses the need for self-assessment of one’s intellectual strengths and limitations, but also an appreciation of the contributions of others [133, 134]. Key strategies for practicing humility include clarity of thinking, open-mindedness, a commitment to mutual learning and ensuring that researchers do not bring a view of themselves as superior to the research team [132]. It is important for trainees to practice humility during research partnerships by acknowledging their positionality and taking the time to learn about the context and culture of an organization or community [132, 135]. These efforts will support collaboration, mutual learning and responsiveness among researchers, communities and health organizations.

Several organizational barriers and facilitators influence trainees’ opportunity and motivation for engaging in IKT or other research partnerships. Included papers reported a number of competing priorities among trainees (e.g., thesis/project completion), knowledge users (e.g., patient care, service delivery, resource use) and university institutions (e.g., timely thesis completion, resource use). Similarly, previous IKT research has illustrated how competing priorities can hinder successful research partnerships [4, 27, 136]. However, this review details specific challenges that trainees encounter with IKT. Individual trainees may have the motivation to engage in research partnerships, but there is often a lack of structures and resources in place to support the IKT process (e.g., funding to support knowledge user engagement). Our findings build on previous IKT recommendations including co-creating value-driven work with a shared purpose, and thereby fostering mutual gain [129, 137]. Further, institutional resources (e.g., funding) and policies are needed to support research partnership approaches for trainees, including flexibility in thesis guidelines, memoranda of understanding between academic and health system institutions, and tangible resources (i.e., guidelines, worksheets) to support co-creation of common goals.

### IKT or other research partnership outcomes

IKT and other research partnership approaches suggest that active engagement of knowledge users throughout the research process increases the use of research in practice and policy [2, 4]. Despite the positive intent of IKT and research partnerships, there has been limited empirical evidence of the effects of IKT on evidence use to inform policy and practice [4]. Building on Gagliardi and colleagues’ scoping review on IKT [4], we organized our outcome findings into three categories of partnership formation. Most papers reported on intermediate outcomes of partnership formation, such as enhanced relevance of research (*n* = 18), and partnership formation outcomes, such as valuing different perspectives (*n* = 13). However, only nine studies reported the use of research in practice/policy as an outcome, which is the primary goal of IKT. Further, over a quarter (*n* = 20/72) did not report outcomes at all. These findings highlight the lack of studies evaluating the impact of the IKT beyond the partnership formation and relevance of research. Efforts are needed to advance the science by moving the field beyond partnership formation studies and understanding the impact of IKT on conceptual, symbolic and instrumental use.

Our findings align with recent calls to advance IKT evaluation, including Kreindler’s [138] work on using a realist evaluation approach to examine how IKT influences the tangible use of evidence in decision-making related to practice and policy. Realist evaluation investigates “what is it about an intervention that works, for whom, and under what conditions’ [139] and is a valuable approach for understand the complexity and nuances of IKT partnerships. Quantitative research methods are also warranted in IKT research, including the application of valid and reliable measurement tools of knowledge user engagement. Several tools exist for patient and public engagement in research that may be useful in examining broader stakeholder engagement (i.e., patients, public, healthcare providers, health system leaders) [140–142]. Trainees involved in IKT research are encouraged to apply these qualitative and quantitative methods to evaluate their IKT approach and contribute to the growing body of evidence on IKT impacts and outcomes [143].

### Summary of implications

Findings from our review address two of the four main goals of scoping reviews [22]: (1) examine the extent, range and nature of the research activity; and (2) identify research gaps in the existing literature. First, this study mapped what is known about trainee-led IKT and partnership research in the literature. Our findings highlight that more formal training efforts may be needed to support skill development for research trainees involved in partnership research. Further, our findings build on previous IKT work highlighting the need for organizational resources and policies to support research partnership approaches.

Second, this review identified research gaps in the existing IKT literature. To advance our understanding of IKT principles and strategies and how trainees partner with knowledge users, we recommend clearly articulating specific activities used in each level of engagement. These details would help trainees to plan knowledge user engagement during their research projects, including the feasibility and utility of different levels of engagement, and would help to understand differences among types of knowledge users. Further, while the included studies described IKT and research partnership approaches, it was not clear why trainees embark on IKT. Additional empirical research is needed to understand the trainees’ motivations for using an IKT approach in their research. This type of research should also aim to understand the unintended consequences of IKT and the power dynamics experienced by trainees and knowledge users, and to identify strategies to promote authentic collaboration, power-sharing and co-creation of knowledge. Lastly, our findings highlight the need for more evaluation of IKT and partnership approaches. We recommend that trainees evaluate their IKT approach and contribute to the growing body of evidence on IKT impacts and outcomes.

### Limitations

Several limitations may affect the interpretation and use of our review findings. First, although we built our search strategy from an umbrella review of IKT and research partnership reviews [144], partnership research is a broad phenomenon. We narrowed our focus to IKT approaches or similar research partnerships that focused on working with knowledge users throughout the health research process. However, IKT is predominantly a Canadian term, and we may have missed papers that described research partnerships differently or in a language other than English. This may be why no papers were identified from lower- and middle-income countries. Second, we only included papers that were written by trainees. Although we conducted a follow-up search of the author’s information to identify trainee status, it is possible that we may have missed papers and did not capture all trainees who have used an IKT or other research partnership approaches in their work. Third, many papers lacked detail on the IKT principles and strategies, which made it challenging to analyse the IKT approach using the WIDER [32] reporting checklist. A specific reporting guideline for this type of work is needed to improve reporting and understanding of the IKT approach, including level-of-knowledge user engagement and IKT strategies. Fourth, we categorized the barriers and facilitators into three levels (individual, interpersonal, organizational) to aid in interpreting how the individual trainee is situated within the academic-health system. These categories are not absolute; many barriers and facilitators exist across the three levels and could be categorized differently depending on the context. Lastly, we found that a quarter of included papers did not evaluate their IKT approach outcome of partnership formation. However, it is important to note that our focus was on the IKT trainee experience and not specifically on evaluation of IKT. These types of evaluation studies may have been published separately and at a later date, as the impact of IKT projects can take years to be fully realized and evaluated.

## Conclusions

This scoping review of 74 papers describes how health research trainees have used an IKT or other research partnership approach in their research. Most trainees have collaborated with knowledge users in the research question development, recruitment and data collection research stages. We identified key barriers and facilitators related to individual, interpersonal and organizational factors. Findings illustrate the importance of having specific knowledge and skills to engage in research partnerships, interpersonal influences of supervisors and knowledge users, and institutional support for a different way of working in graduate and postgraduate work. We also identified enhanced relevance of research findings as an important outcome of IKT trainee research. These review findings can serve as a basis for future reviews and primary research focused on IKT principles, strategies and evaluation. Further, we expect these findings can be used to inform IKT training efforts including trainee guideline development and academic-health system research supports.

## Supplementary Information

**Additional file 1.** Search strategy.

**Additional file 2**. PRISMA_ScR Checklist.

## Data Availability

All data generated or analysed during this study are included in this published article and its additional information files.

## Abbreviations

IKT: Integrated knowledge translation
IKTRN: Integrated Knowledge Translation Research Network
KT: Knowledge translation
COM-B: Capability, Opportunity, and Motivation–Behaviour
TDF: Theoretical Domains Framework
WIDER: Workgroup for Intervention Development and Evaluation Research
JBI: Joanna Briggs Institute
