# Supplementary material for: Trainee-led research using an integrated knowledge translation or other research partnership approaches: a scoping reviews
Source: Health Res Policy Syst. 2021 Nov 2;19:135. doi: 10.1186/s12961-021-00784-0 (PMC8561363; doi:10.1186/s12961-021-00784-0)

Topics: Integrated Knowledge Transfer	Grad/Postgrad Students
**Keywords [ESBSCO operators]**	
("integrated knowledge translation" OR ikt) OR ((engaged OR participatory OR partnership OR collaborative) N2 research) OR "community partnership"	(masters OR graduate OR thesis OR doctora* OR postdoc* OR "post doc*" OR dissertation) W2 (student* OR candidate* OR fellow*)
**CINAHL Headings [EBSCO]**	
(MH "Cooperative Behavior") OR (MH "Collaboration")	(MH "Students+") AND (MH "Education, Graduate+")
**PsycINFO Headings [EBSCO]**	
((DE "Participant Observation") OR (DE "Collaboration" OR DE "Cross Cultural Collaboration")) OR (DE "Cooperation")	((DE "Graduate Students") OR (DE "Postgraduate Students")) OR (DE "Dental Students" OR DE "Medical Students")
**MEDLINE [MeSH] [Ovid]**	
Community-Based Participatory Research/	Students/ AND exp Education, Graduate/
**EMBASE Emtree headings [Elsevier]**	

'knowledge translation'/exp OR 'participatory research'/exp	graduate student'/exp OR 'medical student'/exp OR 'phd student'/exp OR 'postgraduate student'/exp
**Cochrane Library**	
Keywords only	Keywords only
**Sociological Abstracts Headings [Proquest]**	
MAINSUBJECT.EXACT.EXPLODE("Cooperation"))	(MAINSUBJECT.EXACT("Graduate Students") OR MAINSUBJECT.EXACT("Medical Students"))
**Social Services Abstracts [Proquest]**	
MAINSUBJECT.EXACT.EXPLODE("Cooperation"))	(MAINSUBJECT.EXACT("Graduate Students") OR MAINSUBJECT.EXACT("Medical Students"))
**Dissertations & Theses Global [ProQuest]**	
ab("knowledge translation" OR "participatory research" OR "Collaborat*" OR "cooperat*" OR "IKT" OR "integrated knowledge translation" OR "partner*") OR ti("knowledge translation" OR "participatory research" OR "Collaborat*" OR "cooperat*" OR "IKT" OR "integrated knowledge translation" OR "partner*")	ab((masters OR graduate OR doctora* OR postdoc* OR "post doc*" OR student* OR candidate* OR fellow*)) OR ti((masters OR graduate OR doctora* OR postdoc* OR "post doc*" OR student* OR candidate* OR fellow*))

Research	Date Run	Results
research		
(MH "Research personnel+") OR (MH "Research+") OR(MH "Study Design+")	2020-06-24	1207
(DE "Experimentation" OR DE "Debriefing (Experimental)" OR DE "Evidence Based Practice" OR DE "Experiment Controls" OR DE "Experimental Attrition" OR DE "Experimental Design" OR DE "Experimental Ethics" OR DE "Experimental Instructions" OR DE "Experimental Recruitment" OR DE "Experimental Subjects" OR DE "Experimenter Bias" OR DE "Experimenter Expectations" OR DE "Methodology" OR DE "Action Research" OR DE "Animal Research" OR DE "Consumer Research" OR DE "Experimenters" OR DE "Interdisciplinary Research" OR DE "Online Experiments" OR DE "Research Quality" OR DE "Research Setting") OR (DE "Experimental Design" OR DE "Between Groups Design" OR DE "Clinical Trials" OR DE "Cohort Analysis" OR DE "Followup Studies" OR DE "Hypothesis Testing" OR DE "Longitudinal Studies" OR DE "Repeated Measures" OR DE "Retrospective Studies" OR DE "Single-Case Experimental Design")	26-Jun-20	312
exp research/ or exp research design/ exp Research Personnel/	24-Jun-20	86

'research'/exp OR 'methodology'/exp	24-Jun-20	314
Keywords only	24-Jun-20	6
(MAINSUBJECT.EXACT.EXPLODE("Research") OR MAINSUBJECT.EXACT.EXPLODE("Research Design") OR MAINSUBJECT.EXACT("Researchers"))	24-Jun-20	107
(MAINSUBJECT.EXACT.EXPLODE("Research") OR MAINSUBJECT.EXACT.EXPLODE("Research Design") OR MAINSUBJECT.EXACT("Researchers"))	24-Jun-20	45
(ab(health) OR ab(research)) AND (ti(health) OR ti(research))	20-Sep-20	1,161
		full-text filter applied

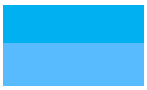
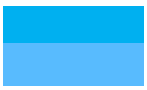
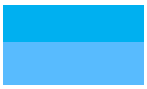
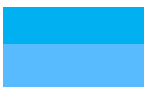
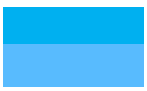

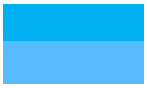
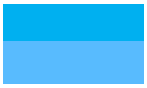
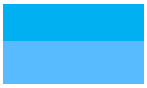
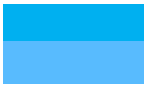
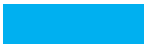

Supplement: Supplementary file 1 — Additional file 1. Search strategy. [file 12961_2021_784_MOESM1_ESM.pdf]
